# Clinical, biochemical and genetic characteristics of patients with argininosuccinate lyase deficiency from a single center cohort in China

**DOI:** 10.1186/s13023-025-04026-2

**Published:** 2025-10-09

**Authors:** Kaichuang Zhang, Deyun Lu, Lili Liang, Yi Yang, Ruifang Wang, Yuning Sun, Zhuwen Gong, Haijuan Zhi, Wenjuan Qiu, Lianshu Han

**Affiliations:** 1 Department of Pediatric Endocrinology and Genetics, Xinhua Hospital Affiliated to Shanghai Jiao Tong University School of Medicine, Shanghai Institute for Pediatric Research, No.1665, Kongjiang Road, Shanghai, 200092 China; 2 Department of Children’s Health, Shanghai Center for Women and Children’s Health, No.339 Luding Road, Shanghai, 200062 China

**Keywords:** Argininosuccinate lyase deficiency, Citrulline, Hyperammonemia, Urea cycle disorders

## Abstract

**Background:**

Argininosuccinate lyase deficiency (ASLD) is a rare autosomal recessive urea cycle disorder (UCD) resulting from mutations in the *ASL* gene. Previous studies of ASLD in patients from China have predominantly been limited to individual case reports, lacking comprehensive cohort study. This study aimed to systematically evaluate the clinical features, biochemical abnormalities, and genetic mutations of Chinese ASLD patients, thereby enhancing the understanding of the unique characteristics of this population.

**Methods:**

We conducted a retrospective analysis of clinical and genetic data from patients diagnosed with ASLD at Shanghai Xinhua Hospital between January 2011 and May 2024.

**Results:**

The cohort consisted of 28 Chinese ASLD patients from Xinhua Hospital. The median age of symptom onset was 18 days (range: 2 days to 6 years). Five patients (17.9%) died within the first year, and 87.0% (20/23) of survivors exhibited developmental delay. Citrulline levels were elevated in all patients. 14 out of 16 (87.5%) who underwent platelet testing demonstrated elevated platelet counts. The median blood ammonia level was 172 µmol/L (range: 9–1911 µmol/L). Early-onset cases had significantly higher ammonia levels than late-onset cases ( *P* < 0.0001). Eight patients underwent liver transplantation, which normalized ammonia levels and liver function but did not prevent developmental delay. Genetic analysis identified 14 novel *ASL* variants.

**Conclusions:**

Our study represents the largest cohort of ASLD patients from China reported to date. Despite active interventions, including liver transplantation, the prognosis remains poor, with a high prevalence of developmental delays. The identification of 14 novel pathogenic variants significantly expands the known mutation spectrum of the *ASL* gene in this population.

## Introduction

Argininosuccinate lyase deficiency (ASLD, OMIM 207900) is a rare autosomal recessive disorder classified within the group of urea cycle defects (UCDs). It results from impaired conversion of argininosuccinic acid into arginine and fumarate, leading to the accumulation of argininosuccinate and citrulline, which serve as key biochemical markers for the disease. The incidence of ASLD varies significantly across regions. In the United States, it is reported at 1 in 218,750, making it the second most common UCD [1]. In contrast, the prevalence in Japan and Korea is much lower, estimated at between 1 in 1,121,000 and 1 in 1,148,000 [2, 3], while in China, the prevalence is reported at 1 in 977,458 [4].

ASLD exhibits a wide phenotypic spectrum, with significant variability in age of onset and severity of symptoms, it is broadly classified into early-onset (EO) and late-onset (LO) forms. EO-ASLD typically presents as neonatal hyperammonemic encephalopathy, a life-threatening condition often associated with poor outcomes despite treatment. In contrast, LO-ASLD manifests later with neurocognitive deficits, learning disabilities, or behavioral abnormalities [5]. The clinical spectrum of ASLD exhibits notably regional variability. Studies from Saudi Arabia, Malaysia, and Italy reported a predominance of EO-ASLD, characterized by severe hyperammonemia and developmental delays. In contrast, cohorts from Austria and the United States are primary LO-ASLD, with many patients maintaining normal ammonia levels yet demonstrating long-term neurocognitive impairments [5–9].

ASLD is caused by mutations in the *ASL* gene. Since the first identification of ASLD over 60 years ago, more than 200 pathogenic variants have been reported, with mutation frequencies varying across populations [10]. ASLD is a panethnic disease with most mutations in a private form and only a few recurring in unrelated patient [11]. The founder mutations have been identified in Arabia (Q354X and Q116X) and Finnish population (R385C) [6, 12, 13]. Despite these advancements, gaps remain in our understanding of ASLD in specific populations, particularly in China, where most reports have focused on individual case studies. This study aims to address this gap by providing a comprehensive analysis of the clinical, biochemical, and genetic characteristics of 28 ASLD patient from a single centre in China.

## Materials and methods

### Participants

The patients who were diagnosed with ASLD at Shanghai Xinhua Hospital in China were enrolled from January 2011 to May 2024. The diagnosis of ASLD was confirmed based on the characteristic clinical symptoms, elevated citrulline levels, and the presence of variants in the *ASL* gene. Clinical data collected for each patient included demographic information (gender, age and birth place), presenting symptoms (such as lethargy, seizures, and developmental delays), and biochemical markers (blood ammonia, citrulline ). Neurological assessments and results from newborn screening were also documented. Written informed consent was obtained from the patients or their guardians. This study protocol was approved by the ethics committee of Xin Hua Hospital Affiliated to Shanghai Jiao Tong University School of Medicine (XHEC-D‐2025‐020).

### Biochemical examination

Blood amino acids, including citrulline were quantified using tandem mass spectrometry (MS/MS, Applied Biosystems API 4000, California, USA) from dried blood spots (DBS). Urine organic acids, including uracil and lactic acid were measured using gas chromatography-mass spectrometry (GC-MS) on a GCMS-QP2010 system (Shimadzu Corporation, Kyoto, Japan). Sample preparation and analysis followed our previous established in-house protocols [14, 15].

### Mutation analysis of the ASL gene

After obtaining informed consent, peripheral blood samples (2 mL) were collected from both the patients and their parents. Genomic DNA was extracted using the TIANamp Blood DNA Kit (Tiangen Biotech Co. Ltd., Beijing, China), following the manufacturer’s protocol. For mutation screening, two different sequencing approaches were employed: targeted clinical exon sequencing was performed on nine patients, and whole-exome sequencing (WES) was conducted on 19 patients. The sequencing data were aligned and analyzed using standard bioinformatic pipelines. The pathogenicity of the identified variants was evaluated based on the American College of Medical Genetics and Genomics (ACMG) guidelines, taking into account variant frequency, conservation, and functional impact [16]. To comprehensively evaluate variant pathogenicity, we first examined their population frequencies in public databases including gnomAD, 1000 Genomes Project, and our internal database. The potential functional impacts were then assessed using multiple computational prediction tools such as SIFT, PolyPhen-2, Mutation Taster. We also analyzed the conservation of affected amino acid residues and their locations within functional domains through multiple sequence alignment. The clinical significance of variants was further evaluated by reviewing previously reported cases in ClinVar database, Human Gene Mutation Database (HGMD), and published literature. Family segregation patterns were investigated through Sanger sequencing validation in probands and their parents.

### Statistical methods

Statistical analysis was conducted using GraphPad Prism 9.0 (GraphPad Software Inc. San Diego, CA, USA). Non-normally distributed continuous variables are expressed using median (min–max). Mann-Whitney U test was used to compare independent variables that are not normally distributed between groups. A P-value of less than 0.05 was considered statistically significant.

## Results

### Patient cohort

A total of 28 Chinese ASLD patients (18 males,10 females) were enrolled in our cohort. During the study period, two additional patients with suspected ASLD were identified but were not included due to incomplete clinical data, specifically lacking conclusive genetic reports required for definitive diagnosis. 8 patients (28.6%) were diagnosed through newborn screening, while the remaining 20 (71.4%) were diagnosed after presenting with clinical symptoms. The probands were geographically distributed across China: 18 cases originated from Eastern China, 5 from Central China, 4 from Southern China, and 1 from Northern China. The median follow-up duration was 1 year, with a range from 1 week to 10 years.

### Clinical manifestations and laboratory data

Of the 28 patients, 14 patients (50.0%) presented with a severe early-onset form, while 12 cases (42.9%) exhibited a late-onset form. The remaining two patients (7.1%) were clinically asymptomatic upon follow-up until 1 year of age and were subsequently lost to further follow-up, thus precluding their definitive long-term clinical classification. The median age of symptom onset was 18 days (2 days to 6 years). Elevated citrulline levels were observed in all the patients, with a median level of 145.4 µmol/L (range: 69.9–401.2 µmol/L). Blood ammonia levels in early-onset patients were significantly higher than those in late-onset patients (*P* < 0.0001). Among 16 patients with platelet data, 14 (87.5%) had elevated platelet counts, with a median of 482 × 10^9/L^ (range: 309–881 × 10^9/L^). Detailed clinical manifestations and laboratory findings are summarized in Tables 1 and 2.

**Table 1:** The clinical data of 28 Chinese patients with argininosuccinate lyase deficiency

Patient	Gender	Age onset/ diagnosis	Follow-Up Duration	Phenotype	MOI NS/CS	NSS	Symptom	LT age	Outcome
1	Male	3d/5m	1y	EO	CS	N/A	Coma	N/A	Death
2	Male	3d/2m	1w	EO	CS	N/A	Coma, feeding refusal	N/A	Death
3	Male	9d/2m	7y	EO	CS	N/A	Lethargy, feeding refusal, seizure	12 m	Alive
4	Male	2d/3m	4 m	EO	CS	P	Lethargy, vomiting	N/A	Death
5	Female	5d/1m	3y	EO	CS	P	Lethargy, feeding refusal	9 m	Alive
6	Male	7d/2m	2y	EO	CS	P	Lethargy, vomiting, seizure	11 m	Death
7	Male	2d/3m	6y	EO	CS	P	Lethargy, feeding refusal	N/A	Alive
8	Male	7d/3m	10y	EO	NS	P	Lethargy, feeding refusal	N/A	Alive
9	Male	2d/5m	1w	EO	CS	N/A	Lethargy, seizure	N/A	Death
10	Female	7d/2m	4y	EO	CS	N/A	Lethargy	2y	Alive
11	Female	13d/2m	1y	EO	CS	P	Coma, seizure	N/A /	Alive
12	Male	11d/2m	1y	EO	CS	P	Lethargy	N/A /	Alive
13	Male	10d/2m	1y	EO	CS	P	Coma, seizure	10 m	Alive
14	Female	23d/2m	7 m	EO	CS	N/A	Lethargy	N/A	Alive
15	Male	17 m/18m	8y	LO	CS	N/A	Coma, intellectual disability	28 m	Alive
16	Male	2 m/4m	9y	LO	CS	N/A	Muscular hypertonia,	N/A	Alive
							intellectual disability		
17	Female	3y/3y	7y	LO	CS	N/A	Seizure, Intellectual disability	N/A	Alive
18	Male	3 m/3m	6y	LO	NS	P	Intellectual disability	N/A	Alive
19	Female	5y/2m	5y	LO	NS	P	Learning disability	N/A	Alive
20	Male	6y/3m	8y	LO	NS	P	Learning disability	N/A	Alive
21	Female	2y/6y	1y	LO	CS	N/A	Global developmental delay	N/A	Alive
22	Male	6y/8y	1y	LO	CS	N/A	Global developmental delay	N/A	Alive
							Learning disability		
23	Male	2 m/3m	1y	LO	CS	N/A	Coma, Motor delay	6 m	Alive
24	Male	20 m/13y	1y	LO	CS	N/A	Intellectual disability, seizure	N/A	Alive
25	Female	1y/2m	1y	LO	NS	P	Motor delay	22 m	Alive
26	Female	8 m/2m	1y	LO	NS	P	Motor delay	N/A	Alive
27	Female	/2m	1y	AS	NS	P	Asymptomatic	N/A	Alive
28	Female	/2m	1y	AS	NS	P	Asymptomatic	N/A	Alive

*D* days, *w* weeks, *m* months, *y* years, *EO* Early Onset, *LO* Late Onset, *AS* Asymptomatic, *MOI* Method of identification, *NS* Newborn Screening, *CS* Clinical Symptoms, *LT* Liver Transplant, *N/A* Not Applicable, *NSS* Newborn Screening Status (*P* Positive).

**Table 2:** Initial biochemical data and imaging examination of 28 Chinese patients with argininosuccinate lyase deficiency

Patient	Ammonia (µmol/L)	ALT (U/L)	AST (U/L)	PLT (10^9)	Cit (µmol/L)	Orotic acid (mmol/molCr)	Uracil (mmol/molCr)	MRI brain	Abdominal Ultrasound	EEG
Reference	9–30	0–75	8–38	100–300	7–35	0–1.5	0–7			
1	530	N/A	N/A	N/A	108.8	0	0	N/A	N/A	N/A
2	604	N/A	N/A	N/A	189.0	N/A	N/A	N/A	N/A	N/A
3	288	198	251	462	108.6	0	0	Normal	Hepatomegaly	Background slowing
4	405	14	22	553	225.6	0	0	Cerebral edema	Normal	Background slowing
5	229	128	82	881	167.5	9.87	14.31	N/A	Normal	Normal
6	560	N/A	N/A	306	401.2	0	54.84	N/A	N/A	N/A
7	196	92	123	498	69.9	8.30	0	Normal	Normal	N/A
8	200	N/A	N/A	N/A	213.0	0	0	N/A	N/A	N/A
9	198	67	43	422	135.7	46.72	0	Subarachnoid hemorrhage	N/A	N/A
10	489	231	168	N/A	118.0	0	High	N/A	N/A	N/A
11	1911	61	57	646	261.0	0	0	Normal	Normal	Background slowing
12	194	27	21	571	130.7	0	0	Normal	Normal	N/A
13	699	216	113	N/A	135.0	N/A	N/A	Normal	N/A	N/A
14	123	54	62	N/A	180.0	0	25.72	N/A	N/A	N/A
15	150	225	81	N/A	175.7	6.16	33.93	N/A	N/A	N/A
16	26	37	80	484	122.7	5.32	39.25	Demyelinating lesions	Normal	N/A
17	22	14	22	274	172.0	25.22	5.34	Normal	Hepatomegaly	Normal
18	138	311	125	352	151.2	0	0	Ventriculomegaly	Hepatomegaly	N/A
19	21	126	103	481	263.2	0	2.54	N/A	N/A	N/A
20	9	27	45	549	111.2	0	0	N/A	N/A	N/A
21	33	13	29	N/A	85.7	0	0	Normal	Normal	Normal
22	10	27	29	N/A	113.2	0	2.84	Abnormal signal (L frontal lobe)	N/A	Epileptiform activity
23	547	151	47	626	258.4	N/A	N/A	N/A	Hepatomegaly	Suspicious sharp waves
24	102	79	82	N/A	145.7	3.23	3.98	Demyelinating lesions	Normal	N/A
25	50	321	129	N/A	145.0	N/A	N/A	Demyelinating lesions	N/A	N/A
26	30	38	39	364	90.0	N/A	N/A	N/A	N/A	N/A
27	65	43	39	339	119.4	0	0	N/A	N/A	N/A
28	21	86	61	N/A	205.0	N/A	N/A	N/A	N/A	N/A

*ALT* alanine transaminase, *AST* aspartate aminotransferase, *PLT* Platelet count, *EEG* Electroencephalogram.

The median age of onset in early-onset patients was 7 days (range: 2–23 days), with a median age at diagnosis of 2 months (range: 2–5 months). Notably, among the 14 early-onset patients, 8 underwent newborn screening; however, 7 of these patients had already developed clinical symptoms by the time the screening results became available. The primary commom neurological symptoms included lethargy (71.4%), coma (35.7%), and seizures (35.7%). Gastrointestinal symptoms, including feeding refusal (35.7%) and vomiting (14.3%), were also noted. Citrulline levels in this group had a median of 151.6 µmol/L (range: 69.9–261 µmol/L). All 14 early-onset patients demonstrated elevated blood ammonia levels, with a median of 346.5 µmol/L (range: 123–1911 µmol/L). Among these 14 early-onset patients, liver function data (ALT and AST levels) were available for 10 individuals; of these 10, 7 (70%) were reported to have liver dysfunction, with median ALT and AST levels of 128 U/L (range: 61–231 U/L) and 113 U/L (range: 43–251 U/L), respectively. Brain MRI findings were normal in five of seven patients, while one exhibited cerebral edema and another subarachnoid hemorrhage. Electroencephalography showed background slowing in all four patients assessed.

Late-onset patients had a median age of onset of 1.3 years (40 days to 6 years) and a median age at diagnosis of 3.5 months (2 months to 13 years). Acute hyperammonemic coma occurred in two cases: Patient 15 at 1 year of age and Patient 23 at 40 days of age. Developmental delay was observed in all patients. Chronic manifestations primarily included intellectual disability (41.7%), learning disabilities (25.0%), motor delay (25.0%) and global developmental delay (16.7%). Epilepsy was reported in one patient (8.3%). The median citrulline level in this group was 145.4 µmol/L (85.7-263.2 µmol/L). Abnormal liver function was reported in 6 of 12 patients, with median ALT and AST levels of 188 U/L (79–321 U/L) and 93 U/L (47–129 U/L), respectively. Elevated blood ammonia levels were found in 4 patients (33.3%), with a median of 144 µmol/L (102–547 µmol/L). Neuroimaging was performed on 6 patients, revealing 2 cases of demyelination, 1 case of ventriculomegaly, 2 cases of abnormal signals in the left frontal lobe, and abnormal signals in the occipital lobes in 1 patient. Hepatic ultrasound revealed hepatomegaly in 3 of 5 patients (60%). Among the late-onset patients who underwent newborn screening (5/12), the median diagnosis age was 3 months, compared to 3.5 years for those who did not undergo screening.

### Treatment and outcome

The majority of patients (24/28) received standard treatment protocols, including a low-protein diet (0.5–1 g/kg/day), arginine supplementation (100–200 mg/kg/day), and sodium benzoate (250–500 mg/kg/day). Four patients (P19, P20, P27, P28), diagnosed through newborn screening with normal ammonia levels and no clinical manifestations, were managed with clinical observation only, without active treatment.

A total of 8 patients (27.6%) underwent liver transplantation, comprising 5 early-onset and 3 late-onset cases. In seven patients, the indication for transplantation was recurrent hyperammonemic crises unresponsive to medical therapy. One patient (P25) underwent transplantation due to progressive neurological deterioration and liver dysfunction. The median age at transplantation was 11 months (range: 6–28 months). For the 6 transplanted patients with follow-up date, the median post-transplant follow-up was 2 years (range: 2 months-6 years). Post-transplant, all patients achieved normalization of blood ammonia levels and liver function tests, even without protein-restricted diets. The median peak blood ammonia levels decreased significantly from 388.5 µmol/L pre-transplant to normal values post-transplant. No infections, graft rejection, or surgical complications were observed during follow-up, except in P6, who died from an infection, and P10, who was lost to follow-up.

Among the cohort, five patients (17.2%) died, all of whom were early-onset cases. Four patients died from hyperammonemic encephalopathy before the age of one year. All the treated patients continued to exhibit varying degrees of developmental delay.

### Gene mutation detection

Genetic test results were available in 28 patients, and they were all diagnosed with two definite disease alleles. Among them, 39 were missense variants (69.6%), 5 were splice (8.9%), 6 were deletion 10.7%), 3 were insertion (5.4%), 2 nonsense variants and 1 duplication. Recurrent variants included c.331 C > T(p.R111W) (5/56), c.437G > A (p.R146Q) (4/56), c.631_647del (p.A211fs*18) (4/56), and c.706 C > T(p.R236W) (4/56). A total of 37 different variants were identified. Fourteen variants were first reported: c.2T > A, c.12 + 1G > T, c.1025T > A, c.1084 C > T, c.1143 + 5G > A, c.1369dupG, c.146T > C, c.258G > C, c.269 C > T, c.301G > C, c.416T > G, c.517 A > T, c.64G > T, exon2-17del. All variants of the *ASL* gene were summarized in Table 3.

**Table 3:** Molecular detection of 28 patients with argininosuccinate lyase deficiency

	Allele1 mother			Allele2 father		
Patient	Nucleotide mutation	Protein alteration	ACMG classifcation	Nucleotide mutation	Protein alteration	ACMG classifcation
1	c.706 C > T	p.R236W	P	**c.1084 C > T**	p.Q362*	P
2	c.1251-1G > A	splicing	LP	**c.12 + 1G > T**	**splicing**	P
3	**c.146T > C**	**p.L49P**	LP	c.557G > A	p.R186Q	P
4	c.889 C > T	p.R297W	P	c.545G > A	p.R182Q	P
5	**c.64G > T**	**p.E22K**	LP	c.337 C > T	p.R113W	LP
6	c.425-426insAGCTCCCAGCT	p.M143Afs*36	P	c.331 C > T	p.R111W	LP
7	c.556 C > T	p.R186W	P	c.437G > A	p.R146Q	P
8	**c.1369dupG**	p.A457Gfs*17	LP	c.437G > A	p.R146Q	P
9	c.631_647del	p.A211fs*18	P	c.889 C > T	p.R297W	P
10	c.631_647del	p.A211fs*18	LP	c.331 C > T	p.R111W	LP
11	**c.1143 + 5G > A**	splicing*(father)	VUS	**c.1143 + 5G > A**	splicing*(father)	VUS
12	c.331 C > T	p.R111W	LP	c.631_647del	p.A211fs*18	P
13	c.425-426insAGCTCCCAGCT	p.M143Afs*36	P	**c.2T > A**	**p.Met1?**	VUS
14	c.437G > A	p.R146Q	LP	c.434 A > G	p.D145G	LP
15	c.206 A > G	p.K69R	LP	c.706 C > T	p.R236W	P
16	c.91G > A	p.D31N	LP	c.706 C > T	p.R236W	P
17	c.1255_1256del	p.L419Vfs*54	P	c.502 C > T	p.R168C	VUS
18	**c.258G > C**	**p.E86D**	P	c.331 C > T	p.R111W	LP
19	c.916 C > T	p.R306W	VUS	c.631_647del	p.A211fs*18	P
20	c.706 C > T	p.R236W	P	c.280 C > A	p.R94C	P
21	c.311 C > T	p.A104 V	LP	**c.1025T > A**	**p.V342E**	LP
22	**c.269 C > T**	**p.T90I**	LP	c.91G > A	p.D31N	P
23	**exon2-17**	**del**	LP	c.331 C > T	p.R111W	LP
24	c.778 C > T	p.L260f	LP	c.425-426insAGCTCCCAGCT	p.M143Afs*36	P
25	**c.416T > G**	p.L139R	VUS	**c.517 A > T**	**p.I173F**	VUS
26	c.719-1G > C		P	c.772G > A	p.E258K	LP
27	**c.301G > C**	**p.G101R**	LP	c.437G > A	p.R146Q	LP
28	c.91G > A	p.D31N	LP	c.544 C > T	p.R182*	LP

Novel variants are shown in bold; *LP* Likely pathogenic, *P* pathogenic, *VUS* variant of uncertain significance

## Discussion

In this study, we present a comprehensive analysis of clinical and genetic data from the largest cohort of ASLD patients from China. This investigation aims to enhance our understanding of the genotypic and clinical characteristics of ASLD in this cohort.

The clinical manifestations of ASLD in our cohort displayed marked variability, consistent with previous reports from other regions. EO cases were associated with severe hyperammonemic encephalopathy, developmental delays, and a high mortality rate. LO cases exhibited varying degrees of developmental delay, intellectual disability, motor delay and global developmental delay. The mortality rate was 17.9% (5/28), which is lower than the 40% reported in European studies but comparable to the rates observed in the UK (15%) and Japan (14.3%) [17–19]. Developmental delays were noted in 21 of the 23 surviving patients. This contrasts with the generally better outcomes reported in Austrian and American cohorts [9, 20]. 65% of Austrian patients and 8 out of 13 American patients achieved normal development, highlighting potential differences in genetic background, diagnostic timing, and treatment strategies. An international study in 2023, involving 60 ASLD patients, found epilepsy in 60% of cases. In that study, early-onset patients primarily exhibited generalized tonic-clonic seizures, while late-onset patients experienced absence seizures [21]. In contrast, epilepsy was observed in only one patient of our cohort. Similarly, a study of an ASLD cohort in Japan reported no cases of epilepsy [17]. In our cohort, 87.5% of the patients exhibited elevated platelet counts, a phenomenon not previously reported in the literature. Endothelial dysfunction has been documented in ASLD patients [22], and we hypothesize that this might contribute to the observed thrombocytosis. These findings suggest potential phenotypic differences between populations from East Asia and Western countries. In our cohort, 9 late-onset patients exhibited normal ammonia levels, however, they still experienced varying degrees of developmental delays. These observations are consistent with reports from other cohorts [6, 9, 13, 17], suggesting that the pathogenic mechanisms underlying neurological damage require further investigation.

Our findings highlight a critical observation regarding the natural course of ASLD. Four late-onset patients (P19, P20, P26, and P27), identified through newborn screening, had normal ammonia levels at diagnosis and did not receive low-protein dietary intervention. However, after a follow-up period of 8 months to 6 years, they all developed various clinical symptoms, challenging the notion that normal initial biochemical parameters predict favorable outcomes. Recent studies have also indicated that movement disorders in argininosuccinic aciduria become more prevalent with age and independent from the age of onset of hyperammonemia [18]. These findings highlight the importance of long-term follow-up.

The effectiveness of newborn screening in ASL disease remains a topic of discussion. In our cohort, eight early-onset patients underwent newborn screening, but seven had already developed clinical symptoms before the screening results became available. This suggests that newborn screening may have limited impact on the diagnosis of early-onset cases. However, among the five late-onset patients, those who underwent newborn screening were diagnosed significantly earlier than those who did not. This finding highlights the utility of newborn screening in facilitating timely diagnosis for late-onset patients. Some studies have also shown that patients identified through newborn screening demonstrate improved neurological outcomes [9, 20, 23]. Therefore, newborn screening appears to be more beneficial for late-onset cases in ASL disease. One notable finding is the elevated citrulline levels in all patients, with no significant difference between the early and late-onset groups. This finding highlights the importance of citrulline as a biomarker in the screening and diagnosis of ASLD. But some findings suggest that relying solely on citrulline as a biomarker for argininosuccinic aciduria screening may result in missed diagnoses [24, 25]. The incorporation of argininosuccinic acid into screening panels may improve detection.

Genotype-phenotype correlation for some of the mutations in our cohort is in agreement with the literature. The mutation R236W (P1,P15,P16,P20) was associated with both early-onset (*n* = 4) [8, 11, 23, 26] and late-onset [27]. Note that one patients with early-onset phenotype carried another nonsense mutation (Q362X) leading to pretermination of the peptide, therefore be expected to affected the protein activity and structural stability seriously. The R111W mutation was observed in both early-onset (P6, P10, P12) and late-onset cases (P18, P23). The R111W codon was located in conserved region 1 of the peptide and close to the candidate proton donor sites [28]. Mutations in these highly conserved residues were likely to have a severe impact on the catalytic function of the protein [8]. Of note, the another allele in the 3 early-onset patients were small del and insert. P18 simultaneously carried a novel mutation (E86D) located at positions 86 with a substitution of another amino acid (aspartic acid) at the exact site. He was diagnosed of newborn screening, then promptly started treatment with low protein diet and arginine, which may partly explain the heterogeneous phenotype of mutations located in the same positions.

The novel mutation L49P was found in a patient (P3) with compound heterozygous state. He experienced the first attack of hyperammonemia at nineth day after birth and remained neurocognitive defects even though with regular treatment. The other mutation (R186Q) identified in this patient had been reported in multiple ethnic groups. This mutation can result in severe early-onset disease as the R186 residue played a crucial role in tetramer formation [3, 8]. A different mutation involved codon 186 (R186W) was previously reported in patient associated with severe neonatal onset form (*n* = 6) [6, 7] or late-onset patients with milder phenotypes (*n* = 3) [6].

Two novel mutations (IVS1 + 1G > T and IVS15-1G > A) were predicted to affect splicing site and associated with severe early-onset phenotype (P2). The novel mutations G101R was found in a asymptomatic patient (P27). G101R involved changes in the protein sequence close to I100, which did not play direct role in substrate binding or catalysis and associated with mild phenotype [11]. Patient 27 had another pathogenic mutation (R146Q), which was previously reported in an early-onset homozygous patient [23]. However, the compound heterozygous mutations G101R and R146Q caused a mild effect in our patient. Patient 19 carried the known severe mutation R306W [11] and the frameshift mutation c.631-647del (p.V211fs*18), the latter of which caused introduction of a premature stop codon, might cause a sever influence on the protein structure and activity. Herein, the compound heterozygous state of R306W and c.631-647del (p.V211fs*18) caused a mild phenotype. We assumed that whether there exsited interallelic complementation as found in other mutations (D87G and Q286R) [28]. Remarkably, it was important to determine correlation between phenotypic consequences and ASL protein levels as well as its enzyme activity. Further study should be conducted to verify our hypothesis.

Uniparental disomy (UPD) leading to autosomal recessive diseases is relatively rare. Whole exome sequencing of patient 11 in our cohort revealed a large number of homozygous variants in the 7q11.21–q36.3 region, with no copy number variation (CNV) abnormalities detected in this region. Sanger sequencing confirmed that the patient’s father was heterozygous for the *ASL* gene c.1143 + 5G > A variant, while the mother was wild-type. These findings suggest the presence of paternal UPD on chromosome 7. Previous reports have also documented ASLD resulting from UPD, but the two previously reported cases were associated with Silver-Russell syndrome [29, 30]. Notably, however, the underlying cause of the disease in those two cases was maternal uniparental disomy. They presented with hyperammonemic encephalopathy at the ages of 1 year and 10 days, respectively, and exhibited features of Silver-Russell syndrome. In contrast, the patient in our cohort had normal intrauterine growth but developed coma and seizures on day 13 of life, with a peak blood ammonia level of 1,191 µmol/L. Following dialysis treatment, the patient’s condition showed improvement.

While hyperammonemia is a hallmark of UCDs, its role in ASLD is inconsistent. EO-ASLD patients often experience severe neonatal hyperammonemia; however, neurological damage persists even after ammonia levels are controlled. Conversely, LO-ASLD patients frequently exhibit significant cognitive impairments despite normal ammonia levels. ASLD patients tend to experience fewer hyperammonemic crises than those with other UCDs, but, they display more severe cognitive impairments, indicating that hyperammonemia alone may not fully explain the neurological damage seen in ASLD [17, 31, 32]. A recent study showed a global beneficial impact of liver transplantion in early-onset ASLD patients [33]. Emerging evidence suggests that the pathogenesis of ASLD is multifactorial, involving not only the toxicity of accumulated argininosuccinic acid and ammonia but also deficiencies in nitric oxide (NO) production, creatine, polyamines, and catecholamines [34–36]. Indeed, NO supplementation has been shown to improve neurological outcomes in these patients [37], highlighting that the current therapeutic focus on merely reducing blood ammonia levels requires urgent reconsideration.

In conclusion, our study provides important insights into the clinical spectrum and management of ASLD in a Chinese cohort. The variability in presentation and outcomes emphasizes the need for early diagnosis and tailored treatment strategies. However, the relatively small sample size, retrospective nature of this study, and the lack of argininosuccinic acid level data limit the generalizability of our findings. Additionally, long-term follow-up data on neurodevelopmental outcomes post-transplantation are needed to better assess the efficacy of liver transplantation in improving quality of life for ASLD patients. Future studies should focus on optimizing NBS protocols, particularly for severe early-onset cases, and exploring novel therapies that can mitigate hyperammonemic crises before irreversible damage occurs.

## Data Availability

The data that support the findings of this study are available from the corresponding author upon reasonable request.
